# Supplementary material for: Anesthetic technique and postoperative pulmonary complications (PPC) after Video Assisted Thoracic (VATS) lobectomy: A retrospective observational cohort study
Source: PLoS One. 2024 Dec 4;19(12):e0310147. doi: 10.1371/journal.pone.0310147 (PMC11616815; doi:10.1371/journal.pone.0310147)
Supplement: S1 Table — (DOCX) [file pone.0310147.s001.docx]

**S1 Table. Year of operation by anesthesia technique**

|  | *Year of operation* | | | | | ***Total*** |
| --- | --- | --- | --- | --- | --- | --- |
|  | 2017 | 2018 | 2019 | 2020 | 2021 |  |
| General alone | 1880 20.7 % | 2001 22.1 % | 1922 21.2 % | 1667 18.4 % | 1592 17.6 % | 9062 100 % |
| GA + Regional | 339 11 % | 526 17.1 % | 647 21.1 % | 738 24 % | 819 26.7 % | 3069 100 % |
| GA + local | 296 17.3 % | 307 17.9 % | 375 21.9 % | 366 21.4 % | 370 21.6 % | 1714 100 % |
| GA + TEA | 157 24.8 % | 136 21.5 % | 134 21.2 % | 125 19.8 % | 80 12.7 % | 632 100 % |
| ***Total*** | 2672 18.5 % | 2970 20.5 % | 3078 21.3 % | 2896 20 % | 2861 19.8 % | 14477 100 % |
| *χ^2^=320.476 · df=12 · Cramer’s V=0.086 · p<0.001* | | | | | | |
