# Supplementary material for: Anesthetic technique and postoperative pulmonary complications (PPC) after Video Assisted Thoracic (VATS) lobectomy: A retrospective observational cohort study
Source: PLoS One. 2024 Dec 4;19(12):e0310147. doi: 10.1371/journal.pone.0310147 (PMC11616815; doi:10.1371/journal.pone.0310147)
Supplement: S2 Table — (DOCX) [file pone.0310147.s002.docx]

**S2 Table. Patient characteristics by group**

|  |  | GA alone  (*n* = 9062) | GA + Regional  (*n* = 3069) | GA + local  (*n* = 1714) | GA + TEA  (*n* = 632) |
| --- | --- | --- | --- | --- | --- |
|  |  | *n* (%) | *n* (%) | *n* (%) | *n* (%) |
| Age | < 65yo | 3211 (35.4) | 1107 (36.1) | 622 (36.3) | 218 (34.5) |
|  | 65 – 74yo | 3289 (36.3) | 1070 (34.9) | 635 (37.0) | 249 (39.4) |
|  | 75 – 84yo | 2401 (26.5) | 841 (27.4) | 443 (25.8) | 156 (24.7) |
|  | 85yo+ | 161 (1.8) | 51 (1.7) | 14 (0.8) | 9 (1.4) |
| Sex | Male | 5279 (58.3) | 1852 (60.3) | 1003 (58.5) | 376 (59.5) |
|  | Female | 3783 (41.7) | 1217 (39.7) | 711 (41.5) | 256 (40.5) |
| Race^1^ | White | 6090 (67.2) | 2282 (74.4) | 1096 (63.9) | 349 (55.2) |
|  | Black/African American | 564 (6.2) | 205 (6.7) | 70 (4.1) | 35 (5.5) |
|  | Asian | 396 (4.4) | 123 (4.0) | 43 (2.5) | 13 (2.1) |
|  | Other | 2012 (22.2) | 459 (15.0) | 505 (29.5) | 235 (37.2) |
| Ethnicity^1^ | Hispanic |  |  |  |  |
| Functional health status^2^ | Independent | 8976 (99.2) | 3051 (99.5) | 1706 (99.6) | 620 (98.4) |
|  | Partially dependent | 71 (0.8) | 16 (0.5) | 6 (0.4) | 10 (1.6) |
|  | Totally dependent | 1 (<0.1) | 0 | 1 (0.1) | 0 |
| ASA class^3^ | 1 = No disturb | 16 (0.2) | 7 (0.2) | 7 (0.4) | 0 |
|  | 2 = Mild disturb | 1750 (19.3) | 622 (20.3) | 342 (20.0) | 83 (13.1) |
|  | 3 = Severe disturb | 6654 (73.4) | 2283 (74.4) | 1244 (72.6) | 454 (71.8) |
|  | 4 = Life threat | 631 (7.0) | 154 (5.0) | 106 (6.2) | 94 (14.9) |
|  | 5 = Moribund | 1 (<0.1) | 0 | 0 | 0 |
| Elective surgery^4^ | Yes | 9062 (100) | 3069 (100) | 1714 (100) | 632 (100) |
| Steroid use for chronic condition | No | 8742 (96.5) | 2947 (96.0) | 1655 (96.6) | 602 (95.3) |
|  | Yes | 320 (3.5) | 122 (4.0) | 59 (3.4) | 30 (4.7) |
| Ascites within 30 days prior to surgery | No | 9059 (100) | 3069 (100) | 1714 (100) | 632 (100) |
|  | Yes | 3 (<0.1) | 0 | 0 | 0 |
| Systemic sepsis within 48 hours prior to surgery | None | 9029 (99.6) | 3047 (99.3) | 1699 (99.1) | 629 (99.5) |
|  | SIRS | 28 (0.3) | 20 (0.7) | 15 (0.9) | 3 (0.5) |
|  | Sepsis | 4 (<0.1) | 2 (0.1) | 0 | 0 |
|  | Septic shock | 1 (<0.1) | 0 | 0 | 0 |
| Ventilator dependent | No | 9061 (100) | 3069 (100) | 1714 (100) | 632 (100) |
|  | Yes | 1 (<0.1) | 0 | 0 | 0 |
|  |  |  |  |  |  |
| Disseminated cancer | No | 8539 (94.2) | 2920 (95.1) | 1614 (94.2) | 586 (92.7) |
|  | Yes | 523 (5.8) | 149 (4.9) | 100 (5.8) | 46 (7.3) |
| Diabetes | No | 7576 (85.6) | 2536 (82.6) | 1420 (82.8) | 520 (82.3) |
|  | Non-insulin | 1074 (11.9) | 394 (12.8) | 200 (11.7) | 75 (11.9) |
|  | Insulin | 412 (4.5) | 139 (4.5) | 94 (5.5) | 37 (5.9) |
| HTN requiring medication | No | 3816 (42.1) | 1303 (42.5) | 732 (42.7) | 299 (47.3) |
|  | Yes | 5246 (57.9) | 1766 (57.5) | 982 (57.3) | 333 (52.7) |
| CHF in 30 days prior to surgery | No | 9001 (99.3) | 3047 (99.3) | 1706 (99.5) | 627 (99.2) |
|  | Yes | 61 (0.7) | 22 (0.7) | 8 (0.5) | 5 (0.8) |
| Dyspnea^5^ | No | 6234 (83.5) | 1837 (81.6) | 1100 (81.8) | 459 (83.2) |
|  | Moderate exertion | 1197 (16.0) | 400 (17.8) | 236 (17.6) | 92 (16.7) |
|  | At rest | 39 (0.5) | 13 (0.6) | 8 (0.6) | 1 (0.2) |
| Current smoker within 1 year | No | 6298 (69.5) | 2096 (68.3) | 1102 (64.3) | 423 (66.9) |
|  | Yes | 2764 (30.5) | 973 (31.7) | 612 (35.7) | 209 (33.1) |
| History of severe COPD | No | 7383 (81.5) | 2392 (77.9) | 1346 (78.5) | 495 (78.3) |
|  | Yes | 1679 (18.5) | 677 (22.1) | 368 (21.5) | 137 (21.7) |
| Dialysis | No | 9033 (99.7) | 3060 (99.7) | 1707 (99.6) | 630 (99.7) |
|  | Yes | 29 (0.3) | 9 (0.3) | 7 (0.4) | 2 (0.3) |
| Acute renal failure^6^ | No | 7468 (100) | 2250 (100) | 1343 (99.9) | 552 (100) |
|  | Yes | 2 (<0.1) | 0 | 1 (0.1) | 0 |
| Year of operation | 2017 | 1880 (20.7) | 339 (11) | 296 (17.3) | 157 (25.8) |
|  | 2018 | 2001 (22.1) | 526 (17.1) | 307 (17.9) | 136 (21.5) |
|  | 2019 | 1922 (21.2) | 647 (21.1) | 375 (21.9) | 134 (21.2) |
|  | 2020 | 1667 (18.5) | 738 (24) | 366 (21.4) | 125 (19.8) |
|  | 2021 | 1592 (17.6) | 819 (26.7) | 370 (21.6) | 80 (12.7) |
|  |  |  |  |  |  |
| BMI |  | 28.1 (*5.9*) | 28.4 (*6.1*) | 28.5 (*5.9*) | 27.9 (*6.0*) |
| Operation time (min)^1^ |  | 178.4 (*77.9*) | 189.6 (*83.1*) | 174.0 (*80.5*) | 184.2 (*89.0*) |

*M*  = mean. *SD* = standard deviation. ^­^

^1^Not one of the risk factors to compute morbidity

^2^Unknown/missing for *n* = 19.

^3^No ASA class assigned for *n* = 29.

^4^ One of the risk factors to compute morbidity, but not included in the statistical model since only elective surgery cases are included.

^5^Missing for *n* = 2861.

^6^Missing for *n* = 2861.
