# Supplementary material for: Anesthetic technique and postoperative pulmonary complications (PPC) after Video Assisted Thoracic (VATS) lobectomy: A retrospective observational cohort study
Source: PLoS One. 2024 Dec 4;19(12):e0310147. doi: 10.1371/journal.pone.0310147 (PMC11616815; doi:10.1371/journal.pone.0310147)
Supplement: S3 Table — (DOCX) [file pone.0310147.s003.docx]

**S3 Table.  ASA physical status classification by anesthesia technique**

|  | *ASA* | | | | | | ***Total*** |
| --- | --- | --- | --- | --- | --- | --- | --- |
|  | 1-No Disturb | 2-Mild Disturb | 3-Severe Disturb | 4-Life Threat | 5-Moribund | None assigned |  |
| GA alone | 16 0.2 % | 1750 19.3 % | 6654 73.4 % | 631 7 % | 1 0 % | 10 0.1 % | 9062 100 % |
| GA + Regional | 7 0.2 % | 622 20.3 % | 2283 74.4 % | 154 5 % | 0 0 % | 3 0.1 % | 3069 100 % |
| GA + local | 7 0.4 % | 342 20 % | 1244 72.6 % | 106 6.2 % | 0 0 % | 15 0.9 % | 1714 100 % |
| GA + TEA | 0 0 % | 83 13.1 % | 454 71.8 % | 94 14.9 % | 0 0 % | 1 0.2 % | 632 100 % |
| ***Total*** | 30 0.2 % | 2797 19.3 % | 10635 73.5 % | 985 6.8 % | 1 0 % | 29 0.2 % | 14477 100 % |
| *χ^2^=141.277 · df=15 · Cramer’s V=0.057 · Fisher’s p<0.001* | | | | | | | |
