# Supplementary material for: Anesthetic technique and postoperative pulmonary complications (PPC) after Video Assisted Thoracic (VATS) lobectomy: A retrospective observational cohort study
Source: PLoS One. 2024 Dec 4;19(12):e0310147. doi: 10.1371/journal.pone.0310147 (PMC11616815; doi:10.1371/journal.pone.0310147)
Supplement: S4 Table — (DOCX) [file pone.0310147.s004.docx]

**S4 Table. Results from linear regression models examining the differences in probability of morbidity/mortality across anesthesia techniques**

|  | **Predicted morbidity** | | | | **Predicted mortality** | | | |
| --- | --- | --- | --- | --- | --- | --- | --- | --- |
|  | **Est** | **exp(Est)** | **95% CI** | ***p*** | **Est** | **exp(Est)** | **95% CI** | ***p*** |
| Intercept | -2.702 | 0.067 | 0.067 - 0.068 | < .001 | -5.430 | 0.004 | 0.004 - 0.004 | < .001 |
| GA + Regional | -0.001 | 0.999 | 0.986 - 1.013 | 0.914 | 0.003 | 1.003 | 0.968 - 1.038 | 0.878 |
| GA + local | 0.019 | 1.020 | 1.002 - 1.037 | 0.028 | -0.027 | 0.974 | 0.932 - 1.018 | 0.237 |
| GA + TEA | 0.058 | 1.059 | 1.031 - 1.088 | < .001 | 0.115 | 1.122 | 1.048 - 1.202 | < .001 |

*Note.* Predicted morbidity and mortality are log-transformed due to skewed distribution. The estimated coefficient (Est) are exponentiated (exp(Ext)) for interpretation
