# Supplementary material for: Anesthetic technique and postoperative pulmonary complications (PPC) after Video Assisted Thoracic (VATS) lobectomy: A retrospective observational cohort study
Source: PLoS One. 2024 Dec 4;19(12):e0310147. doi: 10.1371/journal.pone.0310147 (PMC11616815; doi:10.1371/journal.pone.0310147)
Supplement: Attachment — Submitted filename: Response to reviewers.docx [file pone.0310147.s005.docx]

We thank the reviewers for their comments and suggestions. Please find below our response to their queries:

Reviewer #1: Thank you for letting me review your paper. I appreciate the effort to collect and interpret this large amount of data. I have some questions and concerns. You claim that there is a significant difference in the length of stay between the different groups while there is no difference in the risk of having a postoperative complication defined as you have. My main concern about your conclusions of the results are:

1. During the years of inclusion the practice has changed a lot and you point out that TEA becomes less common while regional anesthesia becomes a lot more used. You include year of surgery as a covariate in the regression model and there is no significance to what year surgery was done but at the same time the differences in the practice is very large as regional anesthesia more than doubled during the 5 year period. This make me question if the impact of time really is insignificant as you interpret it. Maybe I am not good enough on regression models but maybe you could comment on that?

We thank this reviewer for their critique. Incidences of postop complications were 4.0%, 3.4%, 3.8%, 3.7%, and 4.3%, respectively, for years 2017 to 2021. There is no substantial difference in the odds of postoperative complications by year. Model comparisons between the model with surgery year (as presented in the manuscript) and one without surgery year (not included) showed no substantial difference in model fit (Deviance = 0.0064, df = 1, *p* = 0.940), suggesting surgery year did not have substantial influence on the odds of postoperative complications, after controlling for other covariates in the model.

2. Some covariates in the regression analysis is hard to understand why they are included and some could have been included such as pulmonary function FEV1 and DLCO, but maybe they were not available in your register.

We thank this reviewer for their comment. Covariates included in the models were based on published risk factors used by NSQIP to compute predicted postoperative morbidity and mortality. Since, the GA + TEA group had higher probability of morbidity and mortality, we controlled for all these covariates in the regression model. Variables for pulmonary function were not available in NSQIP. We have included this as a limitation of the study. (Page 19 Ln 275)

3. You mention in your discussion that prolonged air-leak is not part of the composite complication endpoint. That would have been interesting as that is the main reason for prolonged LOS in my experience.

We thank this reviewer for their comment. The information regarding air-leak is not available in the NSQIP dataset and this is listed as a limitation of the study. (Page 19 Ln 279-281)

4. The impact of different operating centers are not commented on. As 658 hospitals are contributing to the data there are most likely a big difference in surgical volumes between hospitals which could affect the results. As TEA becomes less common and regional anesthesia goes in the opposite direction you could speculate that a small volume center could be less likely to adopt new routines and in that way TEA might be a confounding factor.

We thank this reviewer for their comment. NSQIP does not provide data on center specific volume and this has been added as a limitation of the study. (Page 19 Ln 278-279)

Reviewer #2: This retrospective cohort study includes a large number of patients and hospitals. The authors analyzed the incidence of post-operative pulmonary complications (PPC) and length of stay (LOS) after lobectomy in video-assisted thoracic surgery (VATS) according to the analgesic technique—epidural, regional, local anesthesia, or none—associated with general anesthesia. They found that between 2017 and 2021, among 15,084 patients from 658 hospitals, 3.5% to 5.2% exhibited a PPC, with a LOS between 3 and 4 days. The subject is interesting as PPC remains a major complication after pulmonary lobectomy. The number of patients is significant, and the data are relatively complete on key elements for analysis. The manuscript is well-written, though a few elements need clarification for the readers as described below:

About PPC criteria: What is the duration of the study period? Are all reintubations within 30 days collected? What criteria are used for pneumonia? Is the diagnosis consistent across different hospitals and based on robust criteria?

We thank this reviewer for their comment. As this reviewer noted in their summary statement above, the study period was between 2017 and 2021. Postoperative respiratory complications are described for 30 days postoperatively and we have added this to the manuscript. (Page7 Ln103)

The codebook indicated that reintubation at or before 30 days post operation was collected – hence we assume that all reintubations within 30 days are collected. Nonetheless, we recognize that it is possible there may be error in the data that we are unable to account for. The criteria is consistent across all centers for all variables and are defined in the NSQIP PUF that accompanies the dataset. For reintubation, criteria used by NSQIP is as follows:

An unplanned intubation must be noted intraoperatively or within 30 days after the primary procedure AND the following criteria, A AND B below:

A. Patient required placement of an endotracheal tube or other similar breathing tube [Laryngeal Mask Airway (LMA), nasotracheal tube, etc.]

AND

B. Patient required ventilator support, which was not intended or planned.

In addition, to ensure the data collected are of the highest quality, the ACS NSQIP has developed a host of different training mechanisms for the Surgical Clinical Reviewers and conducts an Inter-Rater Reliability (IRR) Audit of selected participating sites.

NSQIP uses a fairly robust criteria for pneumonia as outlined below.

The case must meet Radiology (A) criteria AND ONE of the following TWO Signs/Symptoms/Laboratory (B) scenarios as listed below within the 30 days after the primary procedure.

A. Radiology:

ONE chest radiological exam (X-ray or CT)* demonstrating at least ONE of the following:

ο Infiltrate

ο Consolidation

ο Opacity

ο Cavitation

ο Pneumonia, possible, probable, suspicious for pneumonia

OR

o A diagnosis of pneumonia is rendered by an attending physician or advanced provider based on the findings demonstrated on a chest radiological exam (X-ray or CT).

B. Signs/Symptoms/Laboratory:

SCENARIO #1

At least ONE of the following:

o Fever (>380C or >100.40F) with no other recognized cause

o Leukopenia (<4000 WBC/mm3) or leukocytosis (≥12,000 WBC/mm3)

o For adults ≥ 70 years old, altered mental status with no other recognized cause

AND

At least ONE of the following:

o 5% Bronchoalveolar lavage (BAL) -obtained cells contain intracellular bacteria on direct microscopic exam (e.g., Gram stain)

o Positive growth in blood culture not related to another source of infection

o Positive growth in culture of pleural fluid

o Positive quantitative culture or corresponding semi-quantitative culture from minimally contaminated lower respiratory tract (LRT) specimen (specifically, BAL, protected specimen brushing, endotracheal aspirate) or lung tissue specimen. Organisms identified could include bacteria, viruses, or fungi.

B. Signs/Symptoms/Laboratory:

SCENARIO #1

At least ONE of the following:

o Fever (>380C or >100.40F) with no other recognized cause

o Leukopenia (<4000 WBC/mm3) or leukocytosis (≥12,000 WBC/mm3)

o For adults ≥ 70 years old, altered mental status with no other recognized cause

AND

At least ONE of the following:

o 5% Bronchoalveolar lavage (BAL) -obtained cells contain intracellular bacteria on direct microscopic exam (e.g., Gram stain)

o Positive growth in blood culture not related to another source of infection

o Positive growth in culture of pleural fluid

o Positive quantitative culture or corresponding semi-quantitative culture from minimally contaminated lower respiratory tract (LRT) specimen (specifically, BAL, protected specimen brushing, endotracheal aspirate) or lung tissue specimen. Organisms identified could include bacteria, viruses, or fungi.

Is it pertinent to analyze the data for thoracic epidural analgesia (TEA) in 2021? This data comes from such a small portion of the population (2.8%) that it should at least be mentioned in the discussion.

We thank the reviewer for their comment. We agree that a small number of patients get TEA for VATS lobectomy. However, we opted to include all data available for our analyses in order to provide a thorough understanding of the research question. Inclusion of the small proportion of patients that received epidural analgesia does not create any bias in our statistical analysis; in fact, our results demonstrated a larger 95% CI band for the epidural group, as is often reflected by larger standard errors for smaller sample sizes. We have added this to our discussion as mentioned below.

During the five-year study period, the proportion of cases utilizing regional techniques increased, while the percentage of patients receiving TEA decreased from 24.8% of cases in the dataset in 2017 to 12.7% in 2021. (S1 Table) (Page 18 Ln 238-239)

The distribution of analgesia techniques changed during the study period. Are the hospitals the same throughout the period, or is this linked to a center effect by including data in 2021 from new centers with different practices?

We thank this reviewer for their comment. NSQIP does not provide data on center specific volume and this has been added as a limitation of the study. (Page19 Ln 278-279) NSQIP lists the total number of hospitals contributing to the data – 708 hospitals contributed in 2017 while 685 submitted data in 2021. However, there is no information available if the hospitals were same.

PPC results: Please add the PPC incidence for GA + regional anesthesia for comprehensive information for the reader. Lines 216-218: The results are only summarized at the beginning of the discussion part. There is no description, even a short one, and no reference to the additional material in the results section. However, this is an important aspect of the interpretation of data.

Descriptive statistics of postoperative pulmonary complications by anesthetic group are presented in Table 2. A brief description of these incidences is included in the Results section (Page 12 Ln 161-162). We discuss the results of LOS in discussion. Page 17 Ln 223-224. We have also added the decreased utilization of TEA in discussion. Page18 Ln 237-239

LOS results: The effects on LOS are dramatically increased by the use of Poisson regression. It would be useful for the reader to see absolute values of LOS in the different groups with comparisons before regression. The relevance of the LOS difference could be better considered. Line 193: the reference to the table is incorrect.

We recognize the difference between comparing the raw length of stay values and the modeled length of stay difference in a Poisson regression model. Considering that length of stay is a count variable, we opted to utilize Poisson regression model to take into account the unique nature of count data. As presented in the last row in Table 2 (Page 13), the medians and interquartile ranges of length of stay by anesthetic group are available for readers who are interested in the raw length of stay values. As described in Ln 195 on Page 15 we also reference these raw values in the manuscript. We further illustrate the predicted difference in LOS in Figure 3

We illustrate our findings in Figure 3. The x-axis represents the length of stay (in days) for patients who received GA, whereas the y-axis represents the predicted length of stay for patients who received epidural, local, or regional anesthesia. The black reference line reflects the predicted LOS for epidural, local, and regional anesthesia if there was no difference in LOS as compared to the GA group. The average predicted LOS for the epidural group (grey solid line) is above that of the reference line (black solid line), reflecting that patients who received epidural are more likely to have longer LOS as compared to those who received GA. For example, a patient who received GA may have 3 days LOS, whereas the same patient may have >3 days LOS if they had received epidural. This difference is magnified with increased LOS. On the other hand, the average predicted LOS for the local (tan dotted line) and regional (blue dashed line) is below that of the reference line (black solid line), suggesting that patients who received local or regional are likely to have shorter LOS as compared to those who received GA. Of note, the difference in LOS is relatively small (< 1 day difference)

The proportion of ASA 4 patients and operation time are higher in the TEA group. It is possible that TEA was chosen for specific at-risk patients with predicted difficult surgery, or that operation time decreased with increased surgical experience during the study period. Mitigation in the interpretation of the LOS results could be better emphasized in the discussion and conclusion to account for this bias.

We thank this reviewer for the comment. We have added the suggestion to our discussion (Page 19 Ln 265-266) and conclusion. (Page 21 Ln 287-289) Surgical complexity confounding outcomes is also listed as a limitation of the study. Page 19 Ln 275

Line 221 suggests that inadequate analgesia occurs with some types of analgesic techniques. No data on analgesia are presented here, and non-significant results could be due to similar pain scores with different techniques if well-conducted.

We thank this reviewer for the comment. We have added that non-significant results could be due to similar pain scores with different techniques. Page 17 Ln 228-230

Predicted morbidity and mortality scores appear in the statistical analysis method and additional results but are not described or interpreted in the main document, or I did not notice them. Are they still useful to the article, or are they outside the focus of this article?
The typos in the text on lines 184-186 and 82-83 are not consistent.

Results examining the association between probability of morbidity/mortality and anesthetic group are described in Page 15 Ln 209-214 and the corresponding S4 Table. We thank this reviewer for pointing out the typos and these have been corrected.

Please review these points to enhance the clarity and accuracy of your manuscript

Reviewer #3: The authors present a sound written large database analysis on the association of anesthetic techniques with post-surgical pulmonary complications. The paper is well written and the discussion In the discussion, conclusions are drawn that are based on the data. Like all retrospective work, there are limitations to the informative value. The authors found no correlation between the type of anesthesia procedure and respiratory complications. however, they do represent an effect on LOS. This difference may be statistically significant, but a difference of less than one day's stay on average appears to be of little clinical relevance. The authors should discuss this point. It would be helpful to show whether there were fewer very long stays in the group with regional procedures.

We thank this reviewer for their feedback. This comment is similar to one of Reviewer #2’s comments above -

We illustrate our findings in Figure 3. The x-axis represents the length of stay (in days) for patients who received GA, whereas the y-axis represents the predicted length of stay for patients who received epidural, local, or regional anesthesia. The black reference line reflects the predicted LOS for epidural, local, and regional anesthesia if there was no difference in LOS as compared to the GA group. The average predicted LOS for the epidural group (grey solid line) is above that of the reference line (black solid line), reflecting that the patients who received epidural are more likely to have longer LOS as compared to those who received GA. For example, a patient who received GA may have 3 days LOS, whereas the same patient may have >3 days LOS if they had received epidural. This difference is magnified with increased LOS. On the other hand, the average predicted LOS for the local (tan dotted line) and regional (blue dashed line) is below that of the reference line (black solid line), suggesting that patients who received local or regional are likely to have shorter LOS as compared to those who received GA. Of note, the difference in LOS is relatively small (< 1 day difference)
